# Abscisic Acid Refines the Synthesis of Chloroplast Proteins in Maize (*Zea mays*) in Response to Drought and Light

**DOI:** 10.1371/journal.pone.0049500

**Published:** 2012-11-13

**Authors:** Xiuli Hu, Xiaolin Wu, Chaohai Li, Minghui Lu, Tianxue Liu, Ying Wang, Wei Wang

**Affiliations:** 1 Key Laboratory of Physiological Ecology and Genetic Improvement of Food Crops in Henan Province, Henan Agricultural University, Zhengzhou, China; 2 College of Life Science, Henan Agricultural University, Zhengzhou, China; 3 College of Agronomy, Henan Agricultural University, Zhengzhou, China; Kyushu Institute of Technology, Japan

## Abstract

To better understand abscisic acid (ABA) regulation of the synthesis of chloroplast proteins in maize (*Zea mays* L.) in response to drought and light, we compared leaf proteome differences between maize ABA-deficient mutant *vp5* and corresponding wild-type *Vp5* green and etiolated seedlings exposed to drought stress. Proteins extracted from the leaves of *Vp5* and *vp5* seedlings were used for two-dimensional electrophoresis (2-DE) and subsequent matrix-assisted laser desorption/ionization time-of-flight (MALDI-TOF) mass spectrometry (MS). After Coomassie brilliant blue staining, approximately 450 protein spots were reproducibly detected on 2-DE gels. A total of 36 differentially expressed protein spots in response to drought and light were identified using MALDI-TOF MS and their subcellular localization was determined based on the annotation of reviewed accession in UniProt Knowledgebase and the software prediction. As a result, corresponding 13 proteins of the 24 differentially expressed protein spots were definitely localized in chloroplasts and their expression was in an ABA-dependent way, including 6 up-regulated by both drought and light, 5 up-regulated by drought but down-regulated by light, 5 up-regulated by light but down-regulated by drought; 5 proteins down-regulated by drought were mainly those involved in photosynthesis and ATP synthesis. Thus, the results in the present study supported the vital role of ABA in regulating the synthesis of drought- and/or light-induced proteins in maize chloroplasts and would facilitate the functional characterization of ABA-induced chloroplast proteins in C_4_ plants.

## Introduction

The biogenesis of plastids is regulated by external and internal factors, particularly light and phytohormones, such as abscisic acid (ABA) [1]–[3]. ABA is involved in regulating many aspects of plant growth and development, especially in modulating the response to stressful conditions [4], [5]. ABA signal transduction has been extensively studied, and numerous signaling components have been identified, including the chloroplast envelope-localized ABA receptor [6]. Previous reports have shown that some chloroplast proteins, such as the light-harvesting chlorophyll a/b binding proteins and chloroplastic antioxidant defense enzymes, are involved in ABA signal transduction and play a positive role in the response to ABA [3], [7], [8].

Mature chloroplasts are thought to contain approximately 3,000 proteins [9], but the functions of most of these proteins are either unknown or poorly understood. The expression of chloroplast proteins and chlorophyll synthesis are significantly affected in crop plant responses to drought [10]. Recently, many proteins regulated by light have been identified through proteome analysis in green and etiolated maize seedlings [2], [11]. ABA has attracted much research attention as a potentially useful trait in selecting for drought tolerance in crops [12]. However, few comparative proteomic studies have addressed the effect of ABA on the drought- and light-regulation of chloroplast proteins.

The *viviparous*-5 (*vp5*) maize mutant is deficient in ABA biosynthesis, with the first step catalyzed by phytoene desaturase being blocked, which results in the precursor phytoene accumulation and the seedlings photobleaching [13]. The chloroplasts in the *vp5* leaves were destroyed, the thylakoids disappeared, and only vesicles were present [14]. Thus, the *vp5* mutant would be useful to study the effect of ABA on chloroplast protein synthesis in response to endogenous ABA, drought and light.

In the present study, protein synthesis in the leaves of ABA-deficient *vp5* maize mutant and *Vp5* wild-type seedlings under normal light, dim light or drought conditions, were compared with gel-based proteome analysis. As a result, corresponding 13 chloroplast proteins of the 24 differentially expressed protein spots in maize leaves were found to be differentially regulated by drought and light and in an ABA-dependent way.

## Materials and Methods

### Plant Material and Treatments

Maize (*Zea mays* L.) mutant *vp5* and wild-type *Vp5* seedlings were used in this study. The *vp5* mutant is deficient in ABA biosynthesis and has decreased amounts of ABA [15]. Homozygous recessive kernels (*vp5*/*vp5*) lack carotenoids, resulting in white endosperm and embryos, which is easily distinguishable from the yellow, wild-type kernels (*Vp5*/-). Because the recessive mutation is lethal in the homozygous state, it is maintained as a heterozygote. Seeds of *vp5* and *Vp5* plants were obtained by selfing plants grown from heterozygous seeds (Maize Genetics Stock Center, Urbana, IL, USA).

*Vp5* and *vp5* seeds were germinated on moistened filter paper after being surface-sterilized for 10 min in 2% hypochlorite and then rinsed in distilled water. After germination for 2 d, both *vp5* and *Vp5* seedlings were cultured in Hogland’s nutrient solution in a light chamber (day 28°C/night 22°C, relative humidity 75%) under two different radiation conditions: 400 µmol m^−2^ s^−1^ photosynthetically active radiation with a 14/10 h (day/night) cycle and 1 µmol m^−2^ s^−1^ dim light with a 14/10 h (day/night) cycle (for etiolated seedlings). After 2 weeks, the seedlings were subjected to drought treatment by placing them in a –1.0 MPa mannitol solution for 8 h at 28°C under relative humidity 40%. Control seedlings were maintained at 28°C under relative humidity 75%. Subsequently, leaves of treated and untreated seedlings were sampled, immediately frozen in liquid N_2_ and stored at −80°C until analysis. Three or five replicates were performed for each treatment.

### Protein Extraction

An aliquot (0.5 g) of leaf tissue was ground in a mortar in liquid N_2_ into fine powder and then homogenized in 4 ml of the buffer containing 1% SDS, 0.1 M Tris-HCl, pH 6.8, 20 mM dithiothreitol and 1 mM phenylmethylsulfonyl fluoride. The homogenate was centrifuged at 15,000 g for 10 min (4°C). The resulting supernatant was mixed with equal volume of buffered phenol (pH 8.0). Phases were separated by centrifugation at 15000 g for 5 min. The phenol phase was mixed with 5 volumes of methanol containing 0.1 M ammonium acetate overnight (−20°C). Protein was precipitated by centrifugation and washed twice with cold acetone. Air-dried protein pellet was dissolved in 2-DE rehydration buffer (8 M urea, 4% CHAPS, 2% IPG buffer, 20 mM dithiothreitol). Protein extract was clarified by centrifugation prior to 2-DE. Protein was quantified by the Bio-Rad protein assay with bovine serum albumin as a standard.

### Two-dimensional Electrophoresis (2-DE)

Protein was loaded onto an 11-cm linear pH 4–7 strip (GE Healthcare, USA) via passive rehydration overnight [16]. Isoelectric focusing was performed with the Ettan III system (GE Healthcare) at 250 V for 1 h, 1,000 V for 1 h and 8,000 V for 10 h (20°C). Focused strips were equilibrated in buffer I (0.1 M Tris–HCl, pH 8.8, 2% SDS, 6 M urea, 30% glycerol, 0.1 M dithiothreitol) for 15 min and then for another 15 min in buffer II (its composition was the same as buffer I, but with 0.25 M iodoacetamide replacement of dithiothreitol). SDS-PAGE was run using a 12.5% polyacrylamide gel (20×15×0.1 cm). The gels were stained with 0.1% colloidal Coomassie brilliant blue (CBB) G overnight and destained in 10% acetic acid. 2-DE protein profiles were obtained and analyzed by PDQuest v7.1 analysis software to screen differentially expressed protein spots. Protein spots with two-fold variations in abundance were subjected to mass spectrometry (MS) for protein identification.

### Mass Spectrometry and Protein Identification

Differentially expressed protein spots were aseptically removed under a laminar flow hood. Processing of gel plugs, trypsin digestion and matrix-assisted laser desorption/ionization time-of-flight (MALDI-TOF) MS were performed as described previously [16]. The MS/MS data were submitted to Mascot 2.2 (http://www.matrixscience.com, Matrix Science) for peptide mass finger printings against the NCBInr database (http://www.ncbi.nlm.nih.gov/). The taxonomic category was *Zea mays* (452,132 sequence entries in NCBI in December 2011). The search was performed allowing one trypsin miscleavage site, and the peptide tolerance and MS/MS tolerance values were set to 0.8 and 2 Da, respectively. Only significant scores defined by a Mascot probability analysis greater than “identity” were considered for assigning protein identity (http://www.matrixscience.com/help/scoring_help.html#PBM). All of the positive protein identification scores were significant (P<0.05, score>60). Functional categorization of the identified proteins was referred to the annotations in UniProt Knowledgebase (UniProtKB) (http://www.uniprot.org/uniprot).

Subcellular localization of identified proteins was based on the reviewed annotation of identified proteins in UniProtKB and the results of bioinformatics prediction by Plant-mPLoc (http://www.csbio.sjtu.edu.cn/bioinf/plant-multi/) and CELLO (http://cello.life.nctu.edu.tw/).

#### ABA assay

Maize leaves (0.5–1.0 g) were ground in liquid N_2_ by using a mortar, extracted with 2 ml ice-cold 80% methanol containing 1 mM butylated hydroxytoluene to prevent oxidation, and then stored overnight at 4°C. The extracts were centrifuged at 12 000 g for 15 min at 4°C. The pellets were extracted once and stored at 4°C for 1 h. The two resulting supernatants were combined and passed through a C18 Sep-Pak cartridge (Waters, Milford, MA, USA). The efflux was collected and dried in N_2_. The residues were then dissolved in 0.01 M phosphate buffer solution (pH 7.4) and concentrations of ABA were determined in enzyme-linked immunosorbent assay (ELISA).

ABA fractions from C_18_ chromatography were assayed for ABA contents via indirect ELISA [17]. ELISA was performed on a 96-well plate. Each well was coated with 100 µl coating buffer (1.5 g/L Na_2_CO_3_, 2.93 g/L NaHCO_3_, 0.02 g/L NaN_3_, pH 9.6) containing 0.25 µg/ml synthetic ABA-ovalbumin conjugates. The coated plates were incubated for overnight at 37°C. Ovalbumin solution (10 mg/ml) was added to each well of the plates for the purpose of blocking nonspecific binding. After incubation for 30 min at 37°C and washing 4 times with pH 7.4 phosphate buffer plus 0.1% Tween-20, each well was filled with 50 µl of either leaf extracts or ABA standards (0∼2,000 ng/ml dilution range), and 50 µl of 20 µg/ml antibodies against ABA (purchased from China Agricultural University, Beijing, China). The plate was incubated for 3 h at 28°C, and then washed as above. IgG-horseradish peroxidase (Sigma-Aldrich, USA) substrate (100 µl of 1.25 µg/ml) was added to each well and incubated for 1 h at 30°C. The plate was rinsed 5 times with phosphate buffer, and 100 µl color-appearing solution containing 1.5 mg/ml *O*-phenylenediamine and 0.008% H_2_O_2_ was added. The reaction was stopped by adding of 50 µl 6 N H_2_SO_4_ per well when the 2,000 ng/ml standard became a pale color, and the 0 ng/ml standard became a deep color in the wells. Color development was detected using an ELISA Reader (model EL310, Bio-TEK, Winooski, VT) at optical density A_490_. ABA contents were calculated as described previously [18]. The results were the means ± se of at least 4 replicates.

### Chlorophyll Extraction and Calculation

Chlorophyll was extracted as described previously [2]. Fresh leaf slices (0.2 g) were immersed in 15 ml of 95% ethanol for 12 h at room temperature in darkness. Debris in the extract was separated by filtration through filter paper, and then rinsed with 95% ethanol several times to completely extract residual chlorophyll. The clear filtrate was transferred to a brown volumetric flask, and the final volume was brought to 25 ml with 95% ethanol. After thorough mixing, 2 ml of the filtrate was collected and the absorbance was measured at 665 nm and 649 nm using a spectrophotometer. The chlorophyll content was calculated using the following equations: chlorophyll a = 13.95A_665_–6.88A_649_, and chlorophyll b = 24.96A_649_–7.32A_665_. The chlorophyll content in each sample was determined based on three absorbance measurements.

### Statistical Analysis

The results of the ABA and chlorophyll assays were the means of five replicates. Means were compared using one-way analysis of variance and Duncan’s multiple range test at the 5% level of significance.

## Results

### ABA Accumulation and Chloroplast Development in *vp5* and *Vp5* Leaves

**Figure 1:**
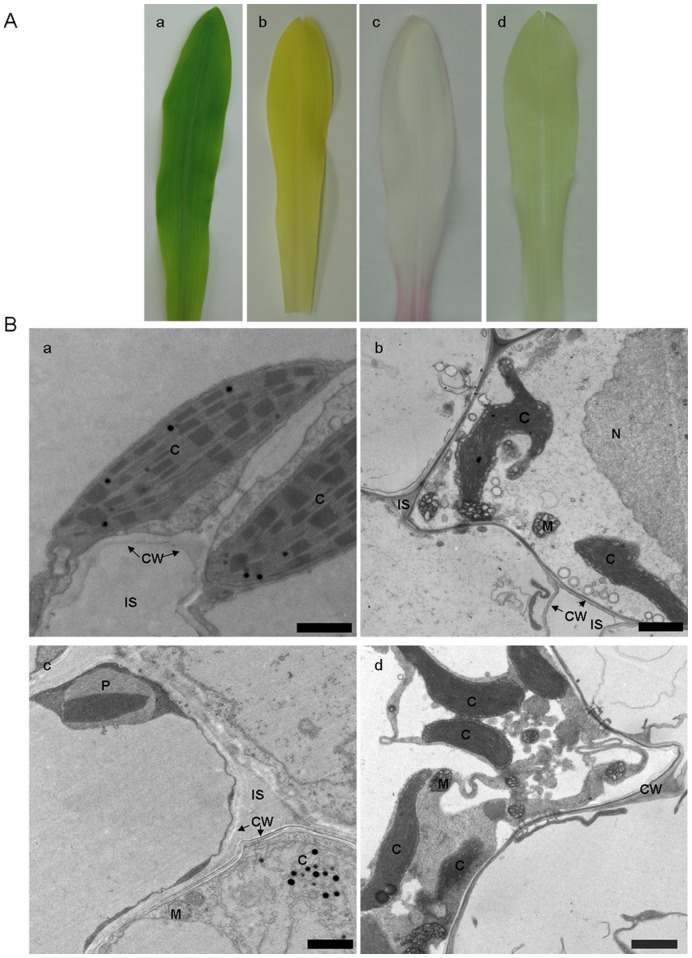
Maize ABA-deficient mutant *vp5* and wild-type *Vp5* leaves under different light conditions. (**A**) Appearance difference of leaves. (**B**) Electron micrographs of leaves. a, *Vp5*, normal light; b, *Vp5*, dim light; c, *vp5*, normal light; d, *vp5*, dim light. All experiments were repeated at least three times. C, chloroplast; CW, cell wall; IS, intercellular space; M, mitochondrion; N, nucleus; P, peroxisome. Bar = 1 µm.

Under normal light, ABA-deficient maize mutant *vp5* seedlings displayed white leaves (Figure 1A, c) compared to green leaves of its wild-type *Vp5* seedlings (Figure 1A, a). Under dim light, *Vp5* seedlings became light yellow (Figure 1A, b), whereas *vp5* seedlings became pale green (Figure 1A, d) and turned white after 8 h of continuous light (data not shown).

Electron microscopic observations revealed distinct differences in the structure of the chloroplasts between *Vp5* and *vp5*. Under normal light, *Vp5* chloroplasts of leaves were typically oval and contained grana consisting of several normally arranged thylakoids (Figure 1B, a). The *vp5* mutation led to internal destruction of chloroplasts and the thylakoids disappeared, with vesicles as the main structures in *vp5* chloroplasts. Besides, ruptures and discontinuities in chloroplast envelope were also observed in *vp5* leaves (Figure 1B, c). Under dim light conditions, the chloroplasts in *Vp5* yellow leaves (Figure 1B, b) and *vp5* pale green leaves (Figure 1B, d) were similar to the chloroplasts of bundle sheath cells, but the former showed obvious deformations and ruptures.

**Figure 2:**
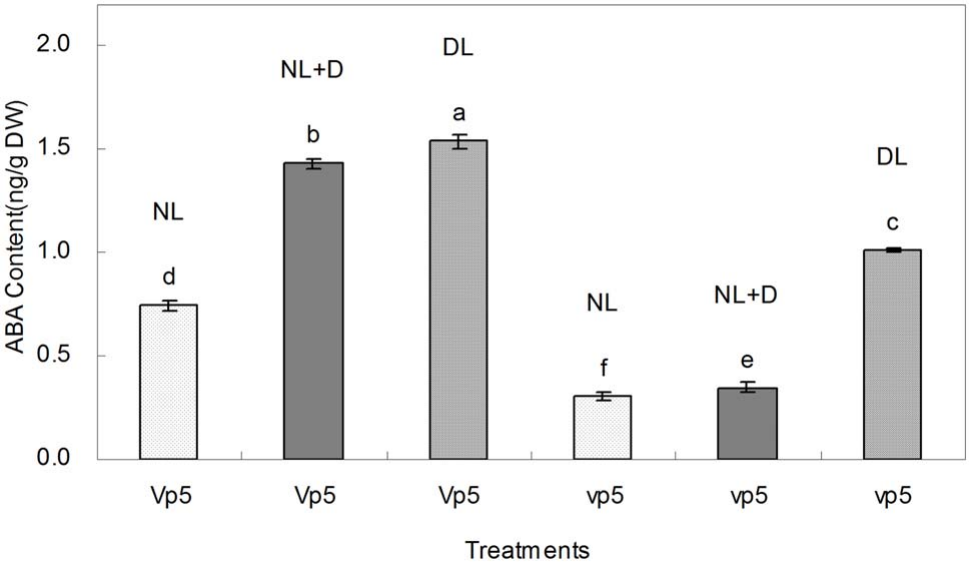
ABA content in maize ABA-deficient mutant *vp5* and wild-type *Vp5* leaves under normal or dim light. NL, normal light (control); DL, dim light; NL+D, drought treatment for 8 h under NL conditions. Values are means ± SE (n = 5).

The ABA content in *vp5* and *Vp5* leaves was measured by ELISA (Figure 2). *Vp5* accumulated more ABA than *vp5*, whether under normal or dim light. Under dim light, however, the increased extent of ABA was significantly higher in *vp5* leaves than in *Vp5* leaves. Under normal light, the ABA increase in *Vp5* leaves induced by drought was much more than that in *vp5* leaves. Taken together, with significant differences in ABA accumulation under light and drought conditions, *Vp5* and *vp5* seedlings were good experimental system to examine the effect of endogenous ABA on the synthesis of chloroplast proteins in response to drought and light.

**Figure 3:**
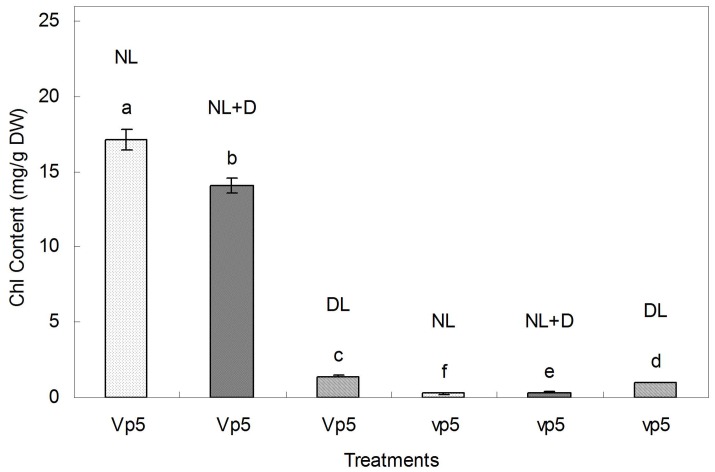
Chlorophyll content in maize ABA-deficient mutant *vp5* and wild-type *Vp5* leaves under normal or dim light . NL, normal light (control); DL, dim light; NL+D, drought treatment for 8 h under NL conditions. Values are means ± SE (n = 5).

In light of the fact that the leaves of ABA-deficient mutant *vp5* turn pale green under dim light, further experiments were performed to determine the effect of ABA accumulation on chlorophyll synthesis. Under normal light, chlorophyll content in *Vp5* leaves was highest (17 mg/g of dry weight) and drought resulted in 17.6% reduction, and chlorophyll content in *vp5* leaves was lower than in *Vp5* leaves (Figure 3). However, under dim light, *Vp5* and v*p5* leaves had comparable chlorophyll contents (Figure 3). These results indicated that light and ABA are the major factors affecting chlorophyll synthesis, while drought has a limited negative effect on chlorophyll synthesis.

### Proteomic Identification of Differentially Expressed Proteins in *Vp5* and *vp5* Leaves in Response to ABA, Drought and Light

**Figure 4:**
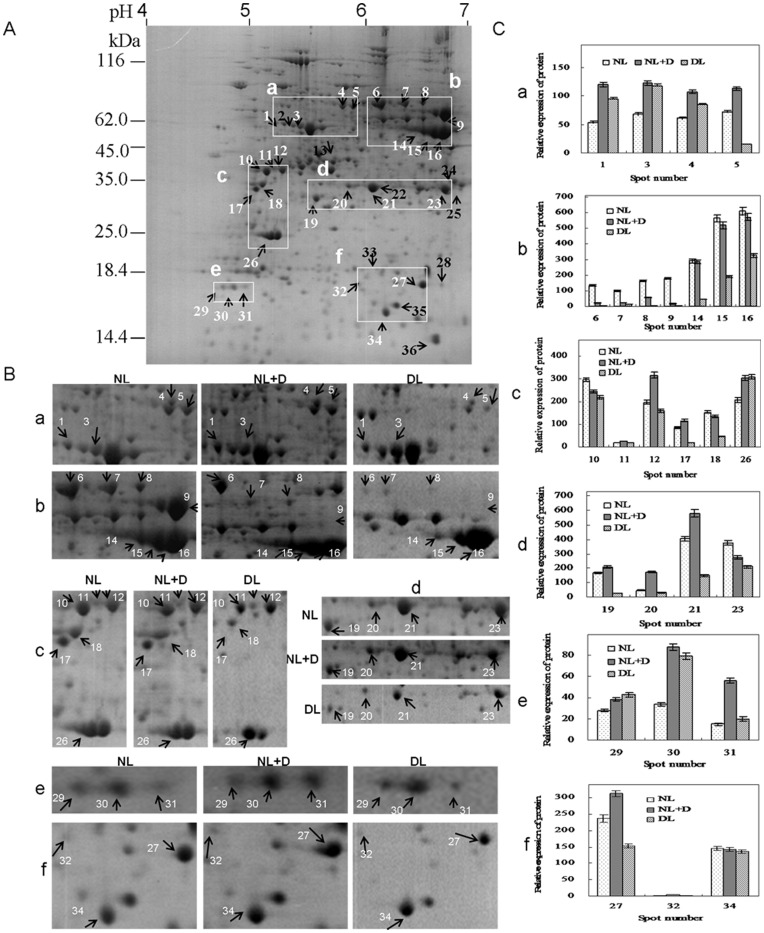
2-DE analysis of differentially expressed leaf proteins in maize *Vp5* seedlings under different treatment. ( A) A representative 2-DE gel of *Vp5* leaves cultured under normal conditions from three biological replicas; (B) Magnified regions of differentially expressed proteins in *Vp5* leaves. NL, normal light (control); DL, dim light; NL+D, drought treatment for 8 h under NL conditions. Protein loads were 800 µg. Gels were CBB G stained. (C) Histograms show the abundance ratio of the identified proteins. Each value represents the average of duplicate 2-DE gels.

**Figure 5:**
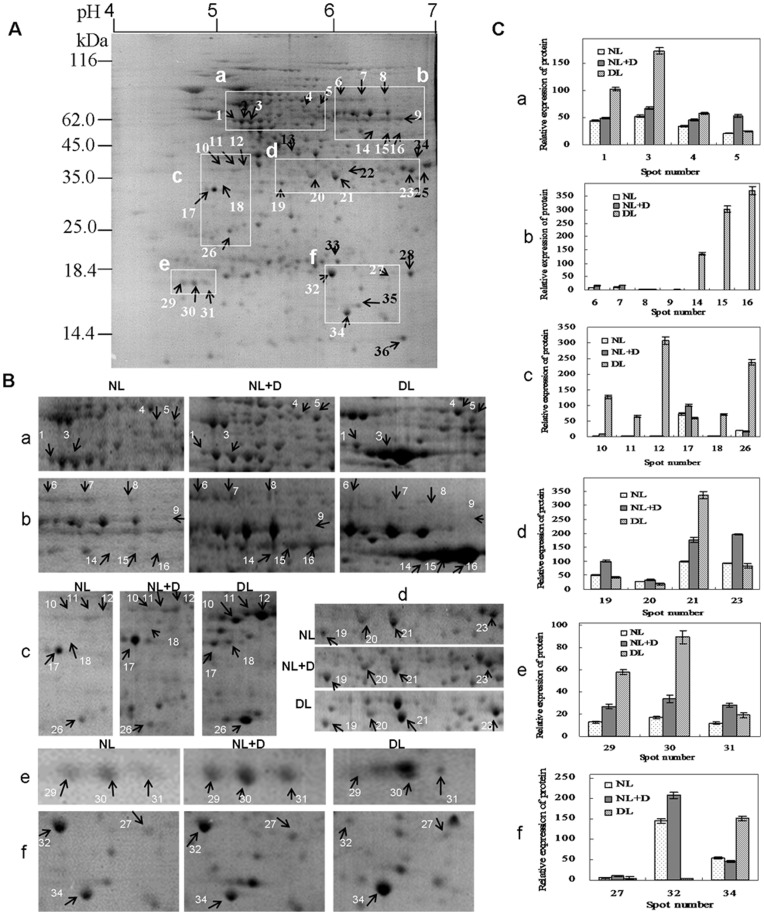
2-DE analysis of differentially expressed leaf proteins in maize ABA-deficient mutant *vp5* seedlings under different treatment. (A) A representative 2-DE gel of *vp5* leaves cultured under normal conditions from three biological replicas; (B) Magnified regions of differentially expressed proteins in *vp5* leaves. NL, normal light (control); DL, dim light; NL+D, drought treatment for 8 h under NL conditions. Protein loads were 800 µg. Gels were CBB G stained. (C) Histograms show the abundance ratio of the identified proteins. Each value represents the average of duplicate 2-DE gels.

Leaf proteins extracted from *Vp5* and *vp5* seedlings cultured under different conditions were resolved by 2-DE. Representative 2-DE gels were presented in Figure 4 (*Vp*5) and Figure 5 (*vp5*) and other 2-DE gels from various treatments were provided in Figure S1, S2, S3, S4. About 450 protein spots were reproducibly detected in each CBB-stained gel, and 36 spots were found to be differentially expressed with 2-fold abundance changes in *Vp5* and *vp5* leaves under different light and drought treatments. The 36 spots were successfully identified using MALDI-TOF MS and involved in ATP synthesis, protein synthesis, chlorophyll synthesis, CO_2_ fixation, gluconeogenesis cycle, antioxidant defense and signal transduction, of which 5 spots (spot 14–16, 18 and 34) belonged to degraded fragments or precursors of ribulose-1,5-bisphosphate carboxylase oxygenase (Rubisco) (Table 1).

**Table 1:** The identification of differentially expressed proteins in maize leaves under different light and drought conditions.

Spot	Protein	UniProt KB Accession	Exp. pI/mass(kDa)	Theor. pI/mass(kDa)	Score^a^	Coverage^b^ (matching petides)	Subcellular Localization			Molecular function
							UniProt KB annotation	Plant-mPLocpredication	CELLOpredication	
1	ATP synthase β-subunit	P20858	5.1/45	5.31/54.04	126	49% (22)	Chloroplast	Chloroplast	Chloroplast	ATP bindingATP synthase activity
2	ATP synthase β-subunit	P19023	5.2/45	6.01/59.10	449	50% (20)	Mitochondrion	Mitochondrion Chloroplast	Mitochondrion	ATP bindingATPase activity
3	ATP synthase β-subunit	P00827	5.3/45	5.31/54.04	512	63% (31)	Chloroplast	Chloroplast	Chloroplast	ATP bindingATP synthase activity
4	Elongation factor 1α	Q41803	5.33/53	5.33/44.57	55	40% (11)	Chloroplast	Chloroplast	Chloroplast	GTP binding, translation elongation factor activity
5	Malic enzyme	Q8W000	5.6/53	6.46/70.74	163	36% (23)	Chloroplast	Chloroplast	Chloroplast	NAD bindingMalate dehydrogenase
6	ATP synthase α-subunit	P05022	6.1/53	5.87/55.71	258	49% (31)	Chloroplast	Chloroplast	Chloroplast	ATP bindingATP synthase activity
7	ATP synthase α-subunit	P05022	6.2/54	5.87/55.71	263	50% (32)	Chloroplast	Chloroplast	Chloroplast	ATP bindingATP synthase activity
8	β-D-glucosidase	P49235	6.6/61	6.72/64.11	168	34% (25)	Chloroplast	Chloroplast	Chloroplast	β-glucosidase activity
9	Rubisco large chain	P00874	6.5/52	6.33/52.70	262	42% (25)	Chloroplast	Chloroplast	Chloroplast Cytosol	Ribulose-bisphosphate carboxylase activity
10	Phosphoribulokinase	B4FQ59	4.9/32	5.84/44.75	419	56% (18)	Chloroplast	Chloroplast	Chloroplast Cytosol	ATP bindingPhosphoribulokinase activity
11	Phosphoglycerate kinase	C0PDB0	5.0/32	5.21/43.20	298	57% (16)	Chloroplast	Chloroplast	Chloroplast Cytosol	Phosphoglycerate kinase activity; Transferase
12	Phosphoglycerate kinase	C0PDB0	5.1/32	5.21/43.20	122	20% (5)	Chloroplast	Chloroplast	Chloroplast Cytosol	Phosphoglycerate kinase activity; Transferase
13	60S ribosomal protein L32 (fragment)	P51421	5.6/41	6.47/4.71	84	66% (2)	Cytosol	None	None	Structural constituent of ribosome
14	Rubisco large chain	P00874	6.4/45	6.33/52.70	127	23% (10)	Chloroplast	Chloroplast	ChloroplastCytosol	Ribulose-bisphosphate carboxylase activity
15	Rubisco large chain	P00874	6.5/45	6.33/52.70	234	36% (22)	Chloroplast	Chloroplast	ChloroplastCytosol	Ribulose-bisphosphate carboxylase activity
16	Rubisco large chain	P00874	6.6/45	6.33/52.70	279	43% (24)	Chloroplast	Chloroplast	ChloroplastCytosol	Ribulose-bisphosphate carboxylase activity
17	ATP synthase β subunit	P00827	4.8/32	5.31/54.04	176	33% (11)	Chloroplast	Chloroplast	Chloroplast	ATP bindingATP synthase activity
18	Rubisco large chain	P00874	4.9/32	6.33/52.70	221	39% (23)	Chloroplast	Chloroplast	Chloroplast Cytosol	Ribulose-bisphosphate carboxylase activity
19	Fructokinase-2	B6TP93	5.3/33	5.34/35.53	425	68% (25)	Chloroplast	Chloroplast	ChloroplastCytosol	Kinase activity
20	ARF-related protein	B6TF60	5.8/30	6.56/22.78	64	15% (2)	Intracellular	Chloroplast	Cytosol Mitochondrial	GTP binding
21	Fructose-bisphosphate aldolase	C0PD30	5.9/30	6.37/38.15	75	10% (3)	Chloroplast	Chloroplast	Chloroplast	Fructose-bisphosphate aldolase activity; lyase
22	Malate dehydrogenase	Q08062	5.9/32	5.77/35.59	134	33% (8)	Cytosol	Chloroplast Mitochondrion	Cytosol Chloroplast	Oxidoreductase, L-malate dehydrogenase activity
23	Glyceraldehyde-3-phosphate dehydrogenase A	P09315	6.8/32	7.00/42.87	186	99% (8)	Chloroplast	Chloroplast	Chloroplast	NAD(P) bindingDehydrogenase activity
24	Glyceraldehyde-3-phosphate dehydrogenase	Q09054	6.9/34	6.40/36.54	130	34% (8)	Cytosol	Cytosol Mitochondrion	Cytosol	NAD(P) bindingDehydrogenase activity
25	Glyceraldehyde-3-phosphate dehydrogenase	Q09054	6.9/34.5	6.40/36.54	263	52% (14)	Cytosol	Cytosol Mitochondrion	Cytosolic	NAD(P) bindingDehydrogenase activity
26	Inorganic pyrophosphatase	B6SQQ0	5.0/22	5.79/31.74	151	40% (15)	Cytosol	Cytosol Chloroplast	Cytosol Mitochondrion	Inorganic diphosphatase activity
27	Protochlorophyllide reductase A	B6TEI7	6.5/18	9.48/41.27	184	27% (6)	Chloroplast	Chloroplast	Chloroplast	Protochlorophyllide reduction
28	Uncharacterized protein	B6TF41	6.7/17	9.75/39.74	114	29% (7)	None	Nucleus	Chloroplast	None
29	2-cys peroxiredoxin BAS1	B6TDA9	4.5/16	5.81/28.15	195	50%(10)	Chloroplast	Chloroplast Cytosol	Chloroplast	Antioxidant activity
30	2-cys peroxiredoxin BAS1	B6TDA9	4.6/16	5.81/28.15	91	40%(4)	Chloroplast	Chloroplast Cytosol	Chloroplast	Antioxidant activity
31	2-cys peroxiredoxin BAS1	B6TDA9	4.7/16	5.81/28.15	229	49%(9)	Chloroplast	Chloroplast Cytosol	Chloroplast	Antioxidant activity
32	Glutathione S-transferase 4	P46420	5.7/18	5.77/24.57	152	48% (15)	None	Cytosol	Cytosol	Glutathione transferase activity
33	Ascorbate peroxidase	B6UB73	5.9/19	5.65/27.39	310	69% (16)	Cytosol	Peroxisome	Cytosol	Peroxidase activity
34	Rubisco large chain	P00874	6.1/16	6.33/52.70	148	26% (14)	Chloroplast	Chloroplast	Chloroplast Cytosol	Ribulose-bisphosphate carboxylase activity
35	Putative NBS-LRR disease resistance protein	Q154F0	6.0/17	5.97/18.10	53	33% (5)	None	Cytosol	Cytosol	ADP binding
36	Abscisic stress ripening protein 2	B4G0Q9	6.5/13	6.15/14.90	131	54% (6)	None	Nucleus	Nucleus	Stress response

a Score is a measure of the statistical significance of a match; ^b^ Percentage of predicted protein sequence covered by matched peptides.

The subcellular localization of these identified proteins was determined based on the experimental results of reviewed accessions in UniProtKB and the prediction results by software Plant-mPLoc and CELLO. Of the 36 identified protein spots, corresponding proteins of 32 spots had definite subcellular localization in UniProtKB: 24 in chloroplast, 7 in cytosol and 1 in mitochondrion (Table 1). In another word, the subcellular localization of these proteins had been determined by experimental methods in previous studies. There still lacked the information on the subcellular localization of the other 4 proteins (spot 28, 32, 35 and 36) in UniProtKB, though software predication suggested the possible subcellular localization such as cytosol (spot 32 and 35) and nucleus (spot 36). Especially, the UniProtKB annotation and software predication on subcellular localization were almost the same for the 24 chloroplast proteins. Therefore, the responses of the 24 chloroplast protein spots (belonging to 13 proteins) to different treatments were summarized in Figure 4 (Vp5) and Figure 5 (vp5), with an emphasis on the comparison between Vp5 and vp5.

ATP synthesis subunit *β* (spot 1 and 3; Figure 4B and 4C, a), phosphoglycerate kinase (PGK) (spot 11 and 12; Figure 4B and 4C, c), 2-Cys peroxiredoxin BAS1 (spot 29–31; Figure 4B and 4C, e) and elongation factor 1α (spot 4; Figure 4B and 4C, a) were significantly up-regulated by both drought and dim light in *Vp5*, while the corresponding spots (Figure 5B and 5C, a, c, e ) were also up-regulated by drought and by dim light in *vp5*, but the increase induced by drought was lower.

Protochlorophyllide reductase A (spot 27; Figure 4B and 4C, f) was significantly up-regulated by drought, but down-regulated by dim light in *Vp5*, while both spots were almost no affected by drought and dim light in *vp5* (Figure 5B and 5C, f).

The intact Rubisco large subunit was highly expressed in *Vp5* under normal light, but degraded under both drought and dim light (spot 14–16, Figure 4B, b; spot 18, Figure 4B, c; spot 34, Figure 4B, f), and the intact Rubisco large subunit was almost undetected. The intact Rubisco large subunit and its degraded fragments or precursors were undetected in *vp5* under normal light and faintly detected under drought stress, whereas these degraded fragments or precursors were strongly expressed under dim light conditions (Figure 5B and 5C, b, c, f).

Fructokinase-2 was slightly increased by drought but markedly reduced by dim light in *Vp5* (spot 19; Figure 4B and 4C, d), while the corresponding spot was markedly increased by drought and reduced by dim light in *vp5* (Figure 5B and 5C, d).

ATP synthesis subunit α (spot 6 and 7; Figure 4B and 4C, b), *β*-glucosidase (spot 8; Figure 4B and 4C, b), glyceraldehyde-3-phosphate dehydrogenase A (spot 23; Figure 4B and 4C, d) and phosphoribulokinase (spot 10; Figure 4B and 4C, c) were significantly reduced by drought and dim light in *Vp5*, while the corresponding spots were markedly increased by drought but reduced by dim light in *vp5* (Figure 5B and 5C, b, d) except that phosphoribulokinase (Figure 5B and 5C, c) was faintly increased by drought but highly increased by dim light.

NADP-dependent malic enzyme (spot 5; Figure 4B and 4C, a) and fructose-bisphosphate aldolase (spot 21; Figure 4B and 4C, d) were markedly increased by drought but decreased by dim light in *Vp5*, while the corresponding spots were markedly increased by drought with a similar increase to *Vp5* by drought, but NADP-dependent malic enzyme (Figure 5B and 5C, a) was little affected by dim light and fructose-bisphosphate aldolase (Figure 5B and 5C, d) was significantly enhanced by dim light in *vp5*.

Besides, Inorganic pyrophosphatase (spot 26), ARF-related protein (spot 20) and Glutathione S-transferase 4 (spot 32) existed in chloroplasts, as suggested only by software predication (Table 1). Inorganic pyrophosphatase (spot 26) was obviously induced by drought and dim light in *Vp5* (Figure 4B and 4C, c), while it was slightly decreased by drought, but increased more by dim light in *vp5* (Figure 5B and 5C, c) than in *Vp5*. ARF-related protein (spot 20; Figure 4B and 4C, d) was significantly up-regulated by drought, but down-regulated by dim light in *Vp5*, while both spots were almost no affected by drought and dim light in *vp5* (Figure 5B and 5C, f). Glutathione S-transferase 4 was easily detectable under normal light and faintly detectable under dim light, and significantly increased by drought in *vp5* (spot 32; Figure 5B and 5C, f), while the corresponding spot was faintly detected and was not changed under different treatments in *Vp5* (Figure 4B and 4C, f).

Taken together, considering the ABA accumulation differences (Figure 2) and the expression patterns of these mentioned-above chloroplast proteins under different treatments (Figure 4 and 5), the roles of ABA, light and drought in regulating the expression of these proteins were summarized in Table 2. For example, drought and light up-regulated the expression of ARF-related protein was in an ABA-dependent way. In total, 13 differentially expressed chloroplast proteins (including 24 protein spots) were found to be up-regulated or down-regulated by ABA.

**Table 2:** The effects of ABA, light and drought on the expression of identified chloroplast proteins in maize.

Protein name (spot)	ABA	Drought	Light
ATP synthase β-subunit (spot 1, 3 and 17)	+	+	−
2-cys peroxiredoxin BAS1 (spot 29–31)	+	+	−
Elongation factor 1α (spot 4)	+	+	−
Phosphoglycerate kinase (spot 11 and 12)	+	+	−
Protochlorophyllide reductase A (spot 27)	+	+	+
Rubisco large chain (spot 9)	+	+	+
ATP synthase α-subunit (spot 6 and 7)	−	−	+
Fructokinase-2 (spot 19)	−	+	+
β-Glucosidase (spot 8)	−	−	+
Glyceraldehyde-3-phosphate dehydrogenase A (spot 23)	−	−	+
Phosphoribulokinase (spot 10)	−	−	+
NADP-dependent malic enzyme (spot 5)	no	+	+
Fructose-bisphosphate aldolase (spot 21)	no	+	+

Note: +, up-regulation;−, down-regulation; no, no effect. Spot 14–16, 18 and 34 were the degraded fragments or precursors of Rubisco large subunit and were not listed in Table 2.

## Discussion

### The Effect of ABA on Chloroplast Development

Chlorophyll locates in the thylakoid sac of chloroplast and associates with specialized proteins in the thylakoid membrane. In higher plants, chlorophyll biosynthesis is a strictly light-dependent multistep process and the light-dependent step is catalyzed exclusively by protochlorophyllide oxidoreductase (POR), which reduces protochlorophyllide to chlorophyllide. In this study, PORA (spot 27, Figure 4) abundance in etiolated maize seedlings was found to be much lower than that in green maize seedlings, which agreed well with PORC expression in *Arabidopsis* [19], [20]. Besides, PORA expression was up-regulated by drought and by light in an ABA-dependent way (Table 2), which was different from the previous result that exogenous ABA treatment inhibits greening and plastid biogenesis in etiolated lupine (*Lupinus luteus* L.) plants under light and promotes the degradation of the light-sensitive POR and reduces the steady-state levels of POR mRNA [21]. Probably, endogenous ABA and exogenous ABA may have different effects on POR expression.

Our results here showed that under normal light, ABA-deficient *vp5* leaves were white, the internal structures of chloroplasts were destructed and the thylakoids disappeared (Figure 1B). However, whether under normal light or dim light, the chlorophyll and ABA contents in *vp5* leaves were significantly lower than its *Vp5* leaves. Obviously, ABA-deficient *vp5* mutation has greatly affected chlorophyll synthesis by affecting the expression of PORA and chloroplast development.

Rubisco is one of the major chloroplast proteins and its regulation occurs at the anabolic level. The inhibition of the syntheses of ABA and Rubisco large subunit affects the chloroplast development [1], [3]. The results in the present study showed that in the ABA-deficient *vp5* mutant, the synthesis of Rubisco large subunit was almost completely inhibited under normal light conditions, but rapidly accumulated with ABA accumulation under dim light. Therefore, light and ABA may regulate chloroplast development by involving in Rubisco synthesis in maize.

### Chloroplast Proteins Regulated by Drought and Light in an ABA-dependent Way

In the present study, 36 differentially expressed leaf protein spots in response to drought and light were identified in *Vp5* and *vp5* maize seedlings, of which 24 spots (belonging to 13 proteins) were definitely localized in chloroplast. Except NADP-dependent malic enzyme (spot 5) and fructose-bisphosphate aldolase (spot 21), the expressions of other 11 chloroplastic proteins were in an ABA-dependent way (Table 2).

Photosynthesis is a key process affected by stress. The expression patterns of most photosynthesis-related proteins are complex under drought stress [22], [23]. For example, drought increases the synthesis of ATP synthase *α*- and *β*-subunits in drought-sensitive wheat [24] and drought-tolerant grass (*Sporobolus stapfianus*) [25], but decreases their synthesis in drought-tolerant wheat [24]. Light up-regulates the synthesis of ATP synthase *β*-subunits with up-regulation by ABA, and light has no effect on *α*-subunits but ABA up-regulated in *Lupinus luteus* [1]. In the present study, ATP synthase *β*-subunit was up-regulated by drought but down-regulated by light with up-regulation by ABA, which was completely contrary to the expression of ATP synthase *α*-subunit (Table 2).

Plant photosynthesis involves transport of electrons in the presence of oxygen; therefore, this process inevitably produces reactive oxygen species (ROS), which are harmful to the cells [26]. 2-Cysteine peroxiredoxins constitute a ubiquitous group of peroxidases that reduce ROS [27] and are up-regulated by ABA and drought [28], [29]. In accordance with these results, our results here showed that 2-Cys peroxiredoxin BAS1 (spot 29–31) was up-regulated by drought and light in an ABA-dependent way.

Translation elongation factor is an essential component involved in protein synthesis. Chloroplast elongation factor is encoded by nuclear DNA [30]. At the mRNA level, elongation factor is rarely regulated by light [31], but down-regulates by salt stress and exogenous ABA [32]. At the protein level, the expression of elongation factor 1α was up-regulated by drought, but down-regulated by light in an ABA-dependent way (spot 4, Figure 4 and 5, Table 2). This discrepancy may be a result of transcription patterns not being directly concomitant with protein expression levels [33].

Pyrophosphate is a key metabolite generated during the activation of a number of polymerization steps, and its removal by inorganic pyrophosphatases is essential to prevent the inhibition of thermodynamically unfavorable reactions [34]. A previous study showed that light induces the biosynthesis of chloroplastic inorganic pyrophosphatase in etiolated maize leaves [35]. In *Nicotiana benthamiana*, the lack of chloroplastic inorganic pyrophosphatase (due to virus-induced gene silencing) increases pyrophosphate level, decreases the contents of chlorophyll, carotenoid and Rubisco large subunit, and has no effect on ABA content under normal conditions, but reduces ABA content and drought tolerance under drought conditions [34]. Likely, the present study indicated that ABA-deficient in maize *vp5* mutant reduced the contents of chlorophyll, Rubisco large subunit, inorganic pyrophosphatase and drought tolerance under drought conditions. Thus, ABA may affect chloroplast performance by regulating inorganic pyrophosphatase.

PGK (spot 11 and 12) is an ATP-generating enzyme that is part of the glycolytic, gluconeogenic and photosynthetic pathways. The chloroplast PGK, which is nuclear encoded, is translated to produce a 50 kDa precursor protein and then processed into the mature 43 kDa PGK in chloroplasts. In the rice scutellum, PGK is stimulated by ABA [36]. In present study, PGK was up-regulated by drought but down-regulated in an ABA-dependent way.

ARF-related protein is a membrane-associated GTPase with a slight similarity to the family of ADP-ribosylation factors, which are a type of small GTP-binding protein. Small GTP-binding proteins regulate a wide variety of intracellular signaling and vesicular trafficking pathways in eukaryotic cells, including plant cells [37]. To our knowledge, the present study was the first to report that light and drought up-regulated the expression of ARF-related protein in an ABA-dependent way.

Proteolysis of Rubisco is a prominent event in the stress-responsive reaction in plants [22]. Table 1 in the present study listed several degraded fragments of the Rubisco large subunit under drought stress. In accordance with previous studies [22], [23], [25], [38], [39], the present study proved that the expression of the Rubisco large subunit was up-regulated light but down-regulated by drought in an ABA-dependent way. Because the degradation of Rubisco is often accelerated by ROS accumulated under drought stress [22], [40], we speculated that H_2_O_2_ accumulation might trigger the drought-induced degradation of the Rubisco large subunit.

Fructokinase catalyzes the phosphorylation of fructose to produce fructose-6-phosphate. A previous study showed that drought significantly up-regulated fructokinase-3 in sunflower [41]. Similarly, our results here showed that both drought and light up-regulated the expression of fructokinase-2 (spot 19) but down-regulated by ABA.

Glutathione S-transferases (GSTs) have been well documented as being involved in diverse aspects of biotic and abiotic stresses, especially detoxification processes. In plants, GST expression is induced by phytohormones, such as ABA and salicylic acid [42], as well as by abiotic stresses, such as drought and salt [43]. Compared to its wild-type, *Arabidopsis* GST-deficient mutant atgstu17 has more tolerant of drought and salt stresses and accumulates higher levels of GSH and ABA [44], [45]. In the present work, we found that glutathione S-transferase 4 (spot 32) was highly expressed only in *vp5* under normal light and drought compared to *Vp5* (Figure 4 and 5), but the reason was unclear. In light of a lower ABA content in *vp5* under normal light (Figure 2), it is suggested that glutathione S-transferase played a negative role in maize drought tolerance in an ABA-dependent way.

In plants, *β*-glucosidases play important roles in key developmental processes, such as growth, pathogen defense and hormone hydrolysis. In *Arabidopsis*, *β*-glucosidase is induced by ABA, salt and drought treatment [46]. In the present study, the expression of *β*-glucosidase (spot 8) was up-regulated by light but down-regulated by drought in an ABA-dependent way.

The glycolytic enzyme glyceraldehyde-3-phosphate dehydrogenase (GAPDH) reversibly converts glyceraldehyde-3-phosphate to 1,3-bisphosphoglycerate via coupling with the reduction of NAD^+^ to NADH. In *Arabidopsis*, the deficiency of plastidial glycolytic GAPDH leads to ABA insensitivity and impairs ABA signal transduction [47]. In potato, drought reduces GAPDH expression [48]. In accordance with these results, the present work also showed that the expression of GAPDH (spot 23) was up-regulated by light but down-regulated by drought in an ABA-dependent way. Recently, GAPDH has been shown to directly interact with an osmotic stress-activated protein kinase in tobacco response to salinity [49]. Therefore, these results suggested that the additional functions of GAPDHs were independent of their catalytic activity and participated in the ABA signal transduction cascades.

Phosphoribulokinase (PRK, spot 10) plays an important role in regulating the flow of sugar through the Calvin cycle. Previous results also showed that PRK synthesis in C_4_ perennial grass species is down-regulated by drought and by ABA treatment [50], but up-regulates by light in rice [51]. Similarly, the present study showed that PRK was up-regulated by light but down-regulated by drought and ABA. PRK catalyzes the phosphorylation of ribulose-5-phosphate to form ribulose-1,5-bisphosphate, which is a key step in the Calvin cycle for CO_2_ assimilation. The down-regulation of PRK indicated that the regeneration of ribulose-1,5-bisphosphate could be inhibited under drought conditions.

In summary, the present study provided basic information that facilitates for functional analysis of maize chloroplast proteins regulated by ABA in response to drought and light. These ABA and drought-responsive proteins are mainly involved in photosynthesis and antioxidant defense, indicating that maintaining photosynthesis and active antioxidant defense mechanisms may play a critical role in the adaptation to drought stress in C_4_ plants.

## Supporting Information

Figure S1 (TIF)

Figure S2 (TIF)

Figure S3 (TIF)

Figure S4 (TIF)
