# Supplementary figures and images for: Abscisic Acid Refines the Synthesis of Chloroplast Proteins in Maize (Zea mays) in Response to Drought and Light
Source: PLoS One. 2012 Nov 13;7(11):e49500. doi: 10.1371/journal.pone.0049500 (PMC3496715; doi:10.1371/journal.pone.0049500)

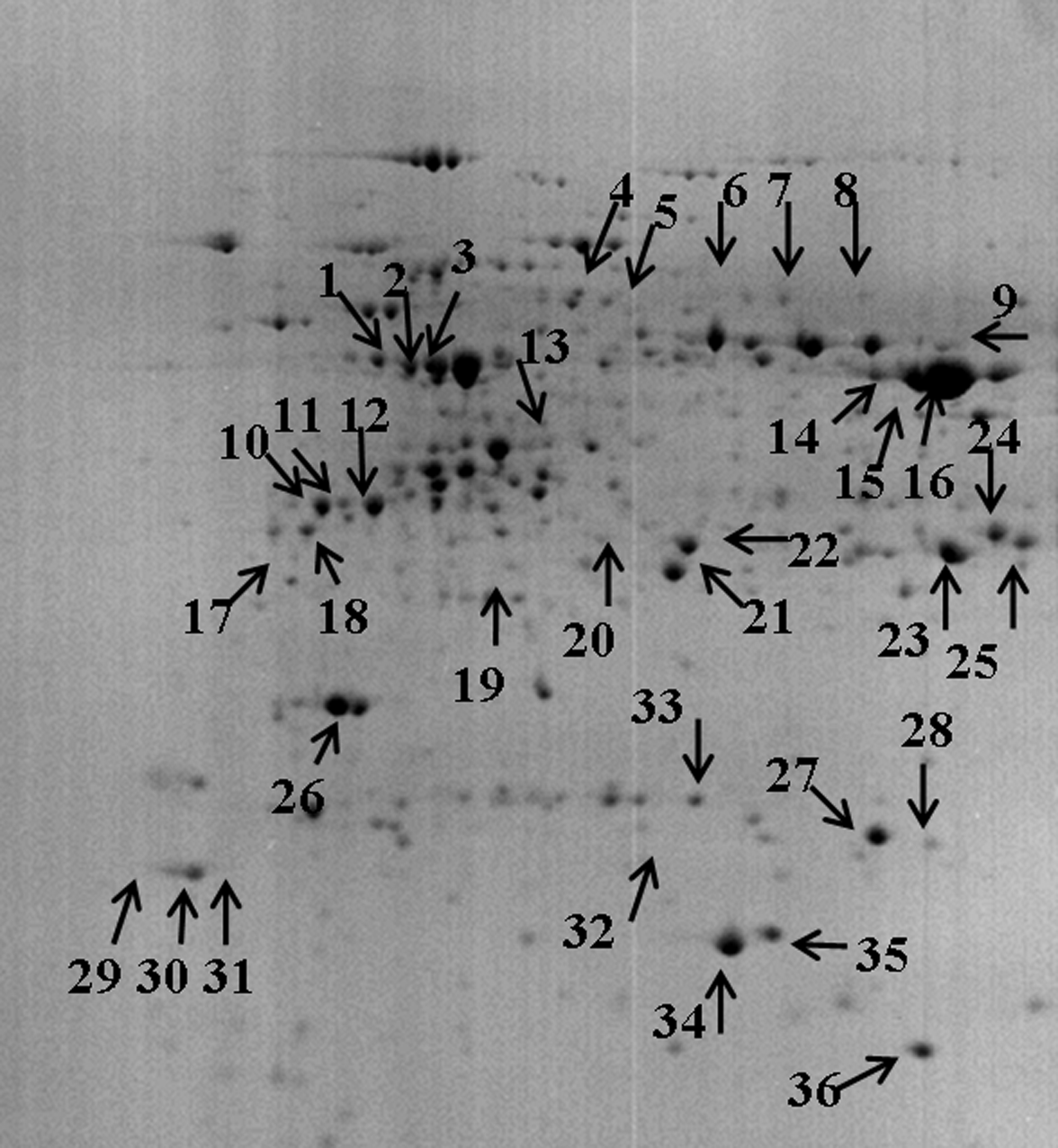

Supplement: Figure S2 — (TIF) [file pone.0049500.s002.tif]

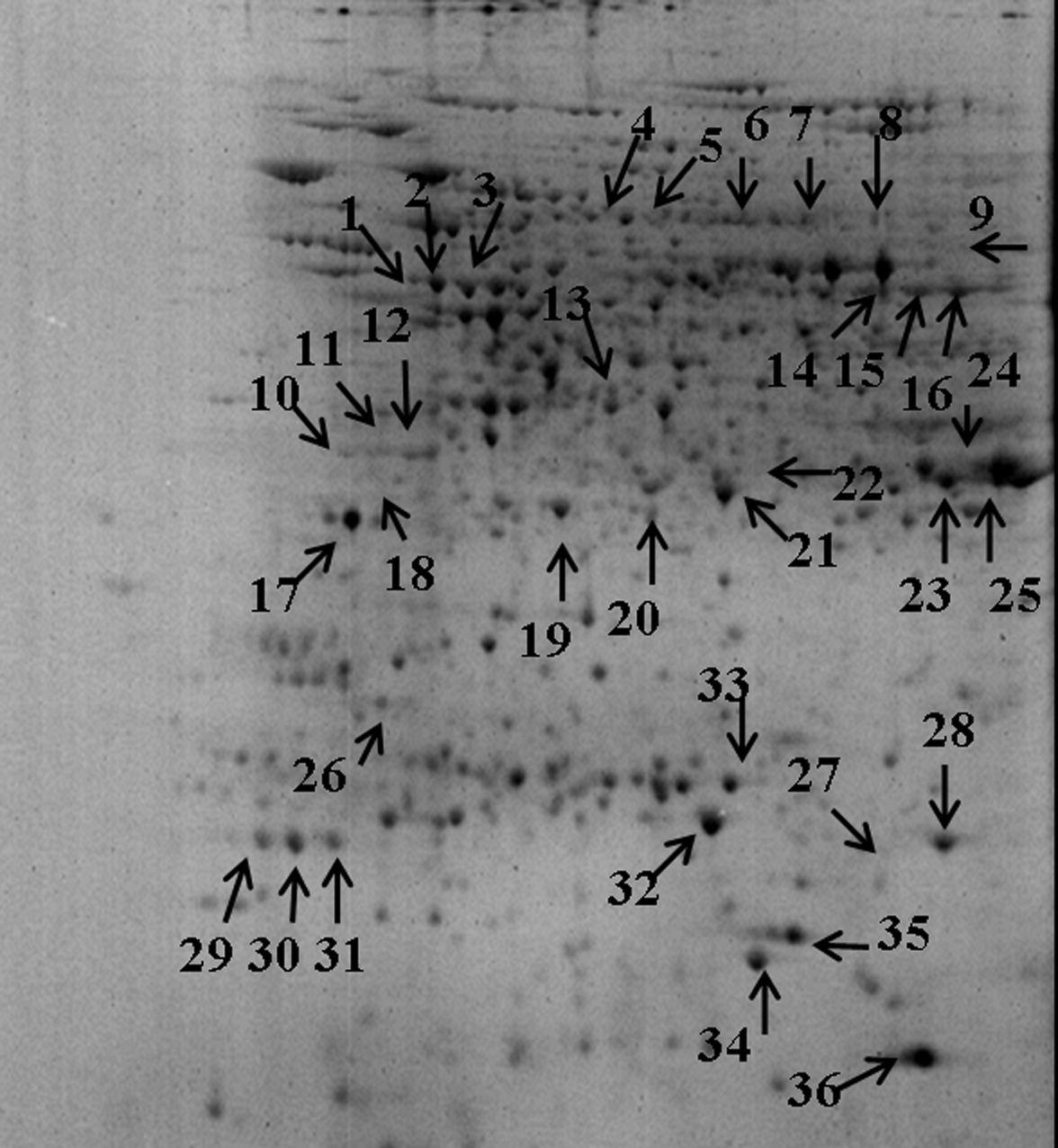

Supplement: Figure S3 — (TIF) [file pone.0049500.s003.tif]
